# Identification of shared gene expression programs activated in multiple modes of torpor across vertebrate clades

**DOI:** 10.1038/s41598-024-74324-5

**Published:** 2024-10-17

**Authors:** Kurt Weir, Natasha Vega, Veronica F. Busa, Ben Sajdak, Les Kallestad, Dana Merriman, Krzysztof Palczewski, Joseph Carroll, Seth Blackshaw

**Affiliations:** 1 Department of Genetic Medicine, Johns Hopkins University School of Medicine, Baltimore, MD USA; 2 Solomon H. Snyder Department of Neuroscience, Johns Hopkins University School of Medicine, Baltimore, MD USA; 3 Genome Biology Unit, European Molecular Biology Laboratories, Heidelberg, Germany; 4 Department of Biology, Johns Hopkins University Krieger School of Arts and Sciences, Baltimore, MD USA; 5 German Cancer Research Center (DKFZ), Heidelberg, Germany; 6 Cell Biology, Neurobiology and Anatomy, Medical College of Wisconsin, Milwaukee, WI USA; 7 Ophthalmology and Visual Sciences, Medical College of Wisconsin, Milwaukee, WI USA; 8 Fauna Bio, Emeryville, CA USA; 9 Biology, University of Wisconsin Oshkosh, Oshkosh, WI USA; 10 Department of Ophthalmology, Gavin Herbert Eye Institute, University of California Irvine, Irvine, CA 92697 USA; 11 Department of Ophthalmology, Johns Hopkins University School of Medicine, Baltimore, MD USA; 12 Department of Neurology, Johns Hopkins University School of Medicine, Baltimore, MD USA; 13 Kavli Neuroscience Discovery Institute, Johns Hopkins University School of Medicine, Baltimore, MD USA; 14 Institute for Cell Engineering, Johns Hopkins University School of Medicine, Baltimore, MD USA; 15 Department of Chemistry, University of California Irvine, Irvine, CA 92697 USA; 16 Department of Molecular Biology and Biochemistry, University of California Irvine, Irvine, CA 92697 USA

**Keywords:** Computational biology and bioinformatics, Evolution, Physiology

## Abstract

Torpor encompasses diverse adaptations to extreme environmental stressors such as hibernation, aestivation, brumation, and daily torpor. Here we introduce StrokeofGenus, an analytic pipeline that identifies distinct transcriptomic states and shared gene expression patterns across studies, tissues, and species. We use StrokeofGenus to study multiple and diverse forms of torpor from publicly-available RNA-seq datasets that span eight species and two classes. We identify three transcriptionally distinct states during the cycle of heterothermia: euthermia, torpor, and interbout arousal. We also identify torpor-specific gene expression patterns that are shared both across tissues and between species with over three hundred million years of evolutionary divergence. We further demonstrate the general sharing of gene expression patterns in multiple forms of torpor, implying a common evolutionary origin for this process. Although here we apply StrokeofGenus to analysis of torpor, it can be used to interrogate any other complex physiological processes defined by transient transcriptomic states.

**Supplementary Information:**

The online version contains supplementary material available at 10.1038/s41598-024-74324-5.

## Introduction

To survive extreme ambient temperatures or food scarcity, some animal species dubbed heterotherms enter torpor, a state of inactivity accompanied by complex physiological changes. Torpid states are marked by suppressed heart rate, metabolic rate, body temperature, oxygen consumption, and blood flow as well as neuroprotective and muscle-protective mechanisms^1–3^, all of which contribute to large energy savings and resilience to muscle wasting and neurological and ischemic damage^2–10^.

Torpor is not limited to a single phylogenetic clade^1–3,5^. For instance, torpor strategies are used by a wide variety of mammals, birds, and reptiles^1–3,5^. Counterintuitively, heterotherms are often more closely related to non-heterotherms within the same clade than they are to other heterotherms^1–3^. It has therefore been suggested that torpor is an ancestral adaptation that has been repeatedly lost and that there are conserved regulatory mechanisms governing torpor between species^1–3^.

There are multiple named variations of torpor including hibernation, aestivation, daily torpor, and brumation. The criteria used to demarcate different forms is inconsistent, but generally include the length of the torpor bout, the season of inactivity, and/or taxonomic separation. For example, bouts of inactivity in daily torpor last less than a day while torpor bouts in hibernation can last weeks^2^. Aestivation is distinguished from hibernation by environmental temperature, with aestivation employed in exceedingly hot periods and hibernation in exceedingly cold conditions^11^. Like hibernation, brumation involves long torpor bouts during the cold season, but the term is applied specifically to reptilian species^5,8,9^. The term “hibernation” is often applied to diverse torpid states. For instance, hibernating bears experience a smaller reduction in body temperature than small mammals during torpor and do not undergo interbout arousals^7^. Similarly, Syrian hamsters store food during torpor rather than store fat as is common among other commonly-studied small mammals^12^. While these terms are segregated, or not, based on these criteria, no definitive answer has been provided regarding how mechanistically similar or dissimilar these torpor strategies are.

Hibernation in small mammals is the most extensively studied form of torpor. It is characterized by long periods of very low activity and body temperature which, unlike other forms of torpor, are interrupted by periodic spikes to euthermic body temperature called interbout arousals (IBA). During this transition from torpor into IBA, gene expression is modified and global transcription as well as energy demands increase^4,10^. Despite the time points demonstrating similar body temperatures, IBA transcriptomes are distinct from euthermic time points^10^. Most early torpor transcriptomic studies have focused on the gene expression differences between euthermia and torpor^9,13–15^. However, due to the variability of activity between stages of torpor, more recent studies include multiple crucially-timed samples throughout torpor, including pre- and post-torpor, IBA, and pre- and post-IBA time points^4,5,7,10,12^. Sampling many time points increases the complexity of analyzing gene expression changes using pairwise comparisons. Various groups have used different approaches to overcome this issue, such as setting the euthermic time point as a single point of comparison or adopting something like a figure-8 approach^4,10^. Often, time points are sampled and sequenced before it is known whether they represent a distinct transcriptomic state, further convoluting comparisons. There is a need for a technique that identifies distinct transcriptomic states and state-specific gene expression programs to simplify and clarify the calculation of stage-specific gene expression.

Independent transcriptomic-based studies have shown consistent gene expression changes during torpor within tissues across species. For instance, genes in the liver involved in carbohydrate catabolism and fatty acid synthesis were broadly downregulated while those related to fatty acid catabolism were broadly upregulated during torpor in the liver of the Chinese alligator (*Alligator sinensis*), Himalayan marmot (*Marmota himalayana*), and grizzly bear (*Ursus arctos horribilis*)^6–8^. Transcriptomic shifts involved in neuroprotection and protection against skeletal muscle atrophy have also been observed across species^4–6^. Recent work has used a gene orthology approach to demonstrate shared molecular mechanisms during torpor among four mammalian species: the arctic ground squirrel (*Urocitellus parryii*), the 13-lined ground squirrel (13LGS, *Ictidomys tridecemlineatus*), the American black bear (*Ursus americanus*), and the Brandt’s bat (*Myotis brandtii)*^1^. However, there have to date been no reported comparisons between torpor transcriptomes in mammal and non-mammal species. Furthermore, comparisons across datasets based on the overlap of lists of significantly differential genes lack the capacity to demonstrate shared global gene expression, which will involve many sub-significant but functionally related changes in gene expression.

We introduce a novel computational pipeline, dubbed StrokeofGenus, to analyze publicly-available torpor bulk RNA-seq datasets. Like prior pipelines, StrokeofGenus identifies orthology across species without the need for a reference genome to enable comparison of gene expression in non-model organisms^16^, but extends its functionality by including non-negative matrix factorization and transfer learning analysis for the discovery of complex patterns of co-expressed genes and the comparison of these patterns across datasets^17,18^. StrokeofGenus is able to identify transcriptomically distinct (and indistinct) phases of torpor and cross-study sharing of torpor gene expression programs. By identifying shared gene expression programs across species, we demonstrate the presence of shared molecular mechanisms directing various forms of torpor, supporting the hypothesis that torpor is a conserved ancestral trait.

## Methods

### Sample collection

Eleven female and four male 13-lined ground squirrels (13LGS, *Ictidomys tridecemlineatus*) were obtained from the University of Wisconsin Oshkosh Squirrel Colony for use in this study. Animals were euthanized by decapitation under isoflurane anesthesia (isoflurane anesthesia was not used for winter torpor decapitations). The frontal cortex, hypothalamus, retina, RPE, and liver were dissected from each of three animals for five physiological states: summer euthermia, prehibernation/room temperature torpor, winter torpor, 3-days-post-arousal euthermia, and 14-days-post-arousal euthermia. Bio Medic Data Systems microchips and/or a FLIR thermal camera pointed at the inner ear were used to determine body temperature and combined with distinct behavioral phenotypes associated with torpor to determine physiological state. The experimental procedures described were approved by the Institutional Animal Care and Use Committee of the Medical College of Wisconsin (AUA00005654) and were performed in accordance with both these guidelines and with the ARVO Statement for the Use of Animals in Ophthalmic and Vision Research, and were reported in accordance with ARRIVE guidelines.

RNAse-free tubes and pipette tips were used. RNAse Zap was used on surfaces, dissection tools, and gloves before and between dissections. Sterile surgery personal protective equipment and tools were used. Tissues were transported in 1 ml of RNAlater per 100 mg of tissue.

### RNA sequencing

Tissue samples were removed from RNAlater and total RNA was isolated with the miRNeasy micro kit with an optional DNase step, per the manufacturer’s protocol (Qiagen, Hilden, Germany). The total RNA was used to generate cDNA libraries with the Illumina TruSeq stranded Total RNA kit and sequenced on an Illumina Nextseq 500 at 50 million reads per sample.

### Dataset download

Fastq files for publicly available torpor datasets in 13LGS (PRJNA418486, PRJNA702062, PRJNA361561), Djungarian hamster (PRJNA743775), Australian central bearded dragon (PRJNA476034), grizzly bear (PRJNA413091), Brandt’s bat (SRP017183), monito del monte (PRJNA416414), Syrian hamster (PRJDB6278), Chinese alligator (PRJNA593416, PRJNA556093), and MCF7 (PRJNA513383) were downloaded from the European Nucleotide Archive.

Sequencing quality was determined using fastqc v0.11.9^19,20^ and adapter sequences removed using trimmomatic v0.39^21^.

### De novo transcriptome generation, gene expression, and proteome inference

The bulk RNA-seq fastq files for each species were input to Trinity v2.13.1^22^ for *de novo* transcriptome assembly using default parameters. Every sample was used for each species, except for 13LGS, where, to save computation time with the high number and redundancy of samples, approximately half of the biological replicates were used (Supplementary Table 3). Gene expression for each sample was calculated using RSEM v1.2.15^23^ and bowtie2 v2.4.1^24^ implemented through the *align_and_estimate_abundance.pl* Trinity script with the --bowtie2_RSEM, --samples_file, --gene_trans_map, and --prep_reference arguments. The *de novo* transcriptome fasta file output by Trinity was used as input for TransDecoder-v5.5.0^25^ to generate predicted proteomes with the “single_best_only” argument so as to produce only one protein sequence for each transcript sequence in the fasta file.

### Orthologue identification

Gene orthology relationships between species were reconstructed from the TransDecoder predicted proteome fasta outputs using OMAStandalone v2.5.0^26^ with the “DoHierarchicalGroups” parameter set to “false” and the “UseOnlyOneSplicingVariant” parameter set to “true” to generate one-to-one gene-level ortholog relationships. Splice files, which are used by OMAStandalone to keep track of splicing variants of the same gene, were generated from the gene_trans_map output of Trinity using a custom script. The yeast proteome from the Orthology Matrix website was used as the out-group in orthology reconstruction in OMAStandalone^27,28^. *M. musculus*, *H. Sapiens*, *D. melanogaster*, and *C. elegans* proteomes were downloaded from the Orthology Matrix website and included in orthology reconstruction as references for the quality of the *de novo* transcriptomes generated in this study.

### Pattern identification and sharing

Gene expression patterns were identified from the RSEM output for each dataset by nonnegative matrix factorization using the R package CoGAPS v3.10.0^17^. For each dataset, a gene expression matrix was constructed by concatenating the gene-level TPM-normalized RSEM output for each sample and fed into CoGAPS. To reduce computation time, we used the *GWCoGAPS* function with the parameter *nSets* = 24 to split pattern finding across twenty-four groups of genes. Pattern markers were calculated for each pattern using the *patternMarkers* function. Shared gene expression patterns across datasets were calculated using the R package ProjectR v1.6.0^18^ with default parameters. Orthology information across species was imported from the OMA Standalone outputs OrthologousMatrix.txt and Map-SeqNum-ID.txt. Only genes with orthologs in both the reference and target datasets were considered.

For some bulk RNA-seq datasets, CoGAPS identified a gene expression pattern with broad, though variable, enrichment across all or many of the samples (Fig. 2D). These patterns represent variable signal in highly expressed genes that is attributable to technical differences such as sequencing depth between samples. Removing technical noise strengthens the identification of variation attributable to biological factors in other patterns (personal correspondence with the creator).

### Prunetree diagram

The prunetree diagram (Fig. S5A) was generated using species names on the TimeTree website^29^.

### Gene set enrichment analysis

Biological process gene ontology (GO) terms were downloaded from MSigD/B^30,31^. For each dataset, all gene names were converted to ortholog human gene symbols and ordered by CoGAPS pattern weights, which are non-negative. We then used the weighted approach implemented in GSEA^32^ via the R package fgsea v1.16.0^33^ with parameter *scoreType* = “pos” to test for enrichment of gene sets.

### Genome download and analysis

The 13LGS genome was downloaded from https://ftp.ncbi.nlm.nih.gov/genomes/all/GCF/016/881/025/GCF_016881025.1_HiC_Itri_2/ and was converted to a form usable by StrokeofGenus with a custom script. Following that, gene expression, proteome inference, ortholog identification, and shared pattern identification were performed as with other datasets. The human genome was downloaded from https://ftp.ensembl.org/pub/release-112/fasta/homo_sapiens/dna/ and gene expression was performed as with other datasets. Ensembl gene names were converted to human OMA names using the Identifier Mapping file available from https://omabrowser.org/oma/current/ and a custom script. Identification of shared gene patterns was performed as with other datasets.

### Data analysis

All data were processed and visualized using R version 4.0.2. Code to recapitulate all analyses is located at https://github.com/vbusa1/StrokeofGenus_manuscript.

## Results

### StrokeofGenus enables cross-comparison of bulk RNA-seq torpor gene expression patterns across non-model species

**Fig. 1:**
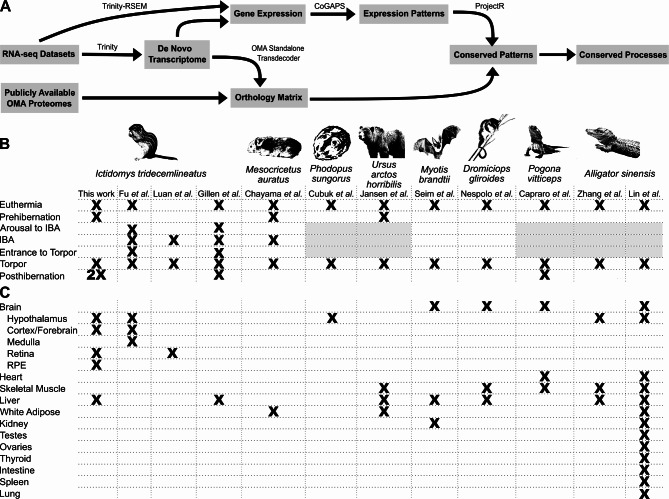
Overview of project design. (**A**) Flowchart for a novel analysis pipeline to reconstruct gene expression patterns and identify shared gene expression across species. The key software used in each step of the analytic pipeline is listed above each arrow. Table of datasets included in the study showing (**B**) time points and (**C**) tissues contained in each dataset. Cells for IBA-associated time points have been greyed out in species that do not undergo IBA.

We established an analysis pipeline able to reconstruct patterns of gene expression within bulk RNA-seq datasets, identify gene orthology across *de novo* transcriptomes, and compare torpor gene expression patterns across non-traditional model organisms (Fig. 1A). First, bulk RNA-seq datasets for each species were fed into the *de novo* transcriptome assembly program Trinity^22^. From here, the pipeline bifurcates. Along one track, we calculated gene expression for each sample using Trinity’s native RSEM capability and the *de novo* transcriptome^23^. Gene expression information for each species was then fed to the non-negative matrix factorization R package CoGAPS^17^ to reveal the underlying structure of the data, including time point and tissue-specific patterns of gene expression. Along the other track, to obtain ortholog information across species, the *de novo* transcriptome was translated using Transdecoder^25^, and each species’ predicted proteome was fed into the orthology program OMA standalone^26^. OMA standalone identified the most similar orthologs for every gene across the species in question. Last, we input the OMA orthology information and CoGAPS-identified gene expression patterns into the transfer learning R package ProjectR^18^ to quantify the presence and strength of gene expression patterns across species.

Multiple approaches have been proposed to compare gene expression across species^1,16,34^ (Table 1). Unlike prior approaches, StrokeofGenus has both an adaptable pipeline, increasing usability, and has demonstrated utility to identify shared gene expression across species. It applies a *de novo* transcriptome assembly approach, enabling the analysis of non-model organisms with poor or missing reference annotations. Finally, StrokeofGenus is the only approach offering both dataset structure detection and orthology identification, and it is unique for its application of transfer learning to directly detect shared gene expression.

**Table 1:** Comparison of usability and utility for approaches to compare gene expression across datasets from different species.

		StrokeofGenus	CoRMAP	Canton et al.	Villanueva-Canas et al.
Usability	Demonstrated across species	✓		✓	✓
	Adaptable pipeline	✓	✓		
Alignment	Reference annotation	✓		✓	✓
	De Novo transcriptome	✓	✓		
Utility	Dataset structure detection	✓		✓	
	De novo orthology detection	✓	✓		
	Expression quantification	✓	✓	✓	
	Quantification of conservation	✓			

StrokeofGenus is available from GitHub and includes a comprehensive vignette. The entire StrokeofGenus pipeline is initiated via the command line and can be completed using only three commands. Although each component of StrokeofGenus was independently developed, the pipeline automates filetype compatibility, file system organization, and output visualization across all tools incorporated in the pipeline, simplifying user experience.

Publicly-available bulk RNA-seq datasets are available studying different forms of dormancy in species from multiple clades^35–37^. To identify shared patterns of gene expression throughout dormancy within vertebrate species, we selected a shortlist of eleven datasets in eight species with overlapping time points and tissues^4,5,7–10,12–15,35^ (Fig. 1B, C). The datasets represent both mammals and reptiles that undergo variations of torpor including hibernation, daily torpor, brumation, and aestivation. The sampled tissues were enriched for central nervous system tissues and metabolically important tissues such as the liver. In addition, to further facilitate intra-species comparisons using StrokeofGenus, we generated a new 13LGS RNA-seq dataset that includes tissues for which data are publicly available in three other 13LGS hibernation datasets^4,10,35^.

Between 7,578 and 19,422 orthologous genes were identified between all species comparisons (Supplementary Table 1). The number of genes reconstructed by Trinity is driven by the number of sequencing reads provided to the program^38^. Accordingly, larger numbers of genes were reconstructed for species with more samples (Supplementary Table 1). Species with greater numbers of reconstructed genes also had more orthologs identified across comparisons than those with fewer reconstructed genes, although this also appears to be impacted by evolutionary distance (Supplementary Table 1).

### Nonnegative matrix factorization identifies tissue- and time point-specific patterns of gene expression

To simultaneously uncover the distinct transcriptomic states and state-specific gene expression of the publicly-available hibernation RNA-seq datasets, StrokeofGenus applies the matrix factorization tool CoGAPS. CoGAPS deconvolves the patterns of coexpression that cumulatively compose the variation within a transcriptomic dataset. Each learned pattern represents phenomena that direct gene expression in a sample, including technical artifacts or biological processes (e.g. tissue type, age, or torpor state). A weight is calculated for each gene for each pattern, with a high weight indicating the gene’s expression greatly contributes to the pattern. Samples also receive a weight for each pattern, with a high weight meaning the pattern is driving a lot of expression in that sample. To attribute functionality to a pattern, we must rely on available metadata. For example, we infer that a pattern enriched in torpid samples over euthermic samples is a torpor pattern. Gene expression associated with that pattern represents torpor-specific gene expression programs.

**Fig. 2:**
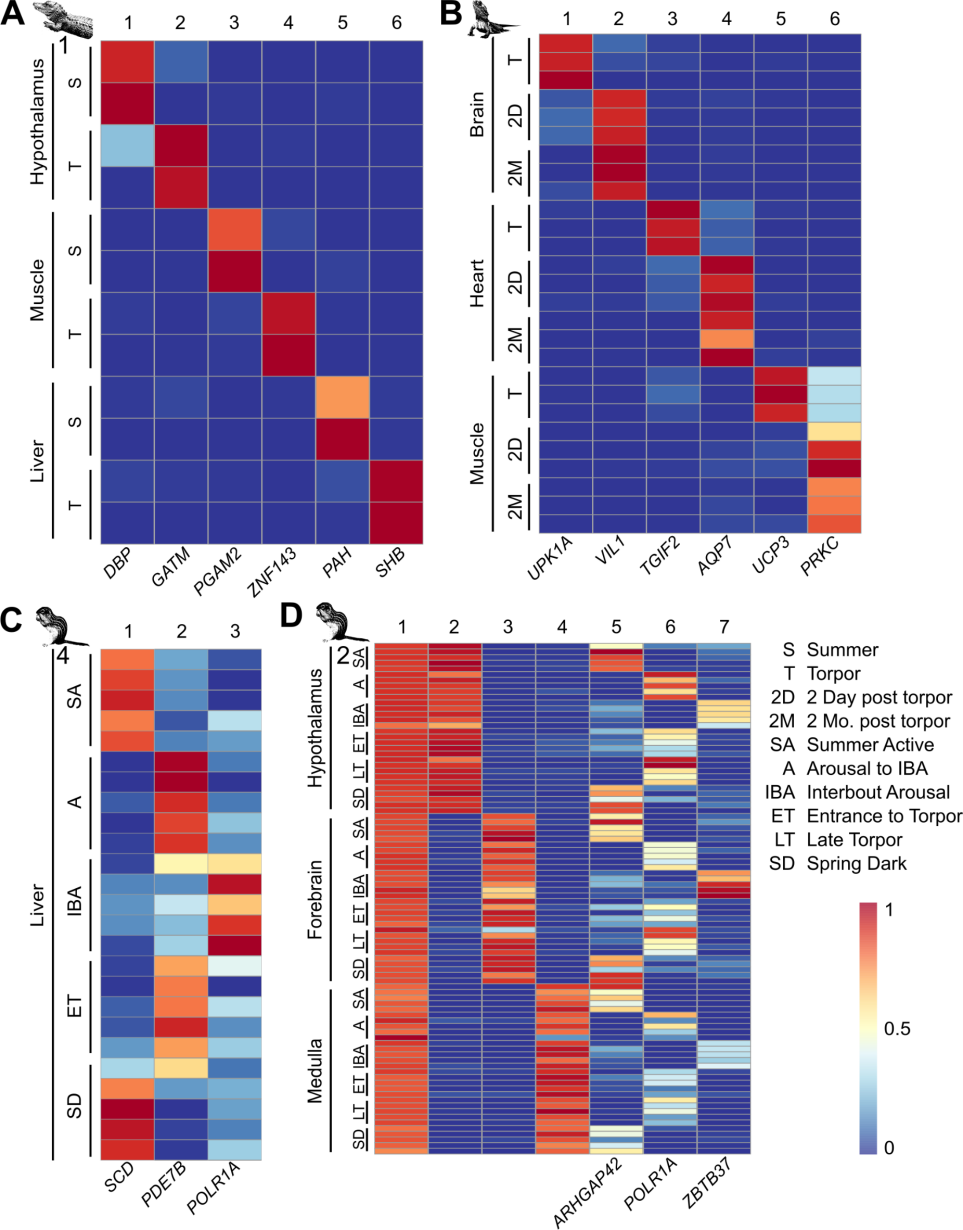
Identification of tissue and time point-specific gene expression patterns in torpor datasets. Heatmaps of tissue/time point-specific gene expression patterns in the (**A**) Chinese alligator dataset 1, (**B**) bearded dragon dataset, (**C**) 13LGS 4 dataset, and (**D**) 13LGS 2 dataset. Each row is a sample and each column is a CoGAPS-derived pattern. Select pattern-specific genes that align with prior analyses are arrayed next to the relevant patterns. All figure panels colored on the same scale with blue representing low pattern weight and red representing high pattern weight.

For almost all the datasets analyzed, CoGAPS was able to identify tissue and/or time point-specific patterns of gene expression (Fig. 2, S1). All datasets produced time point-specific patterns, except the monito del monte dataset, which only produced tissue-specific patterns (Fig. S1D). CoGAPS was also able to deconvolve which time points within each dataset represented distinct transcriptomic states. We directed CoGAPS to identify between two and ten patterns for each dataset’s gene expression matrix. The results for each number of patterns were visualized as a heatmap showing the enrichment for each pattern in each sample, which were used to identify the tissue and time point-specificity of each pattern and optimize pattern number. At low pattern numbers, samples were separated by tissue (Fig. S2A). As the number of patterns increased, time point and tissue/time point-specific patterns emerged (Fig. 2A). To determine how many patterns to derive from a dataset, we set the cutoff for hibernation-related patterns as the maximum number for which each pattern showed tissue and/or time point specificity but not sample specificity. If the number of patterns increased beyond this point, samples from the same time point and tissue would separate into sample-specific patterns (Fig. S2B). We reasoned that only patterns with greater signal than sample-specific gene expression noise were biologically meaningful and useful for the purposes of discovery.

After optimizing the number of found patterns, we determined that some samples obtained from apparently distinct phases of hibernation actually represent equivalent transcriptomic states. For instance, the period immediately following exit from torpor was not transcriptomically distinct from an euthermic time point further temporally separated from torpor in the Australian bearded dragon (Fig. 2B). We also found that the time points immediately preceding and following IBA— Arousal to IBA and Entrance to Torpor— are not distinct from Late Torpor in 13LGS (Fig. 2D). Our results corroborate the original publications’ findings, which were identified using principal component analysis (PCA) and random forest clustering techniques^4,5,10^, respectively. Our results demonstrate that the principal transcriptomic states characterizing hibernation in small mammals are euthermia, torpor, and IBA (Fig. 2C, D). Therefore, for following analyses, samples from Summer Active, Prehibernation, Posthibernation, and Spring Dark time points are considered euthermic and patterns found in those samples are considered euthermic patterns. Similarly, Arousal to IBA, Entrance to Torpor, and Late Torpor samples are considered torpid samples and patterns in those sample are considered torpid patterns.

Tissue and time point specificity manifested differently across datasets. In some datasets, individual patterns show combined tissue and time point specificity (Fig. 2A, B). In other datasets, separate tissue-specific patterns were discovered along with distinct time point-specific patterns that were shared across tissues (Fig. 2D, S2A). We observe that the latter case occurred in 13LGS 2, a dataset composed of closely-related tissues, whereas the former occurred in datasets containing more distantly-related tissues. This is likely because more closely-related tissues share essentially the same gene expression changes while more distantly-related tissues have distinct gene expression programs during torpor.

**Fig. 3:**
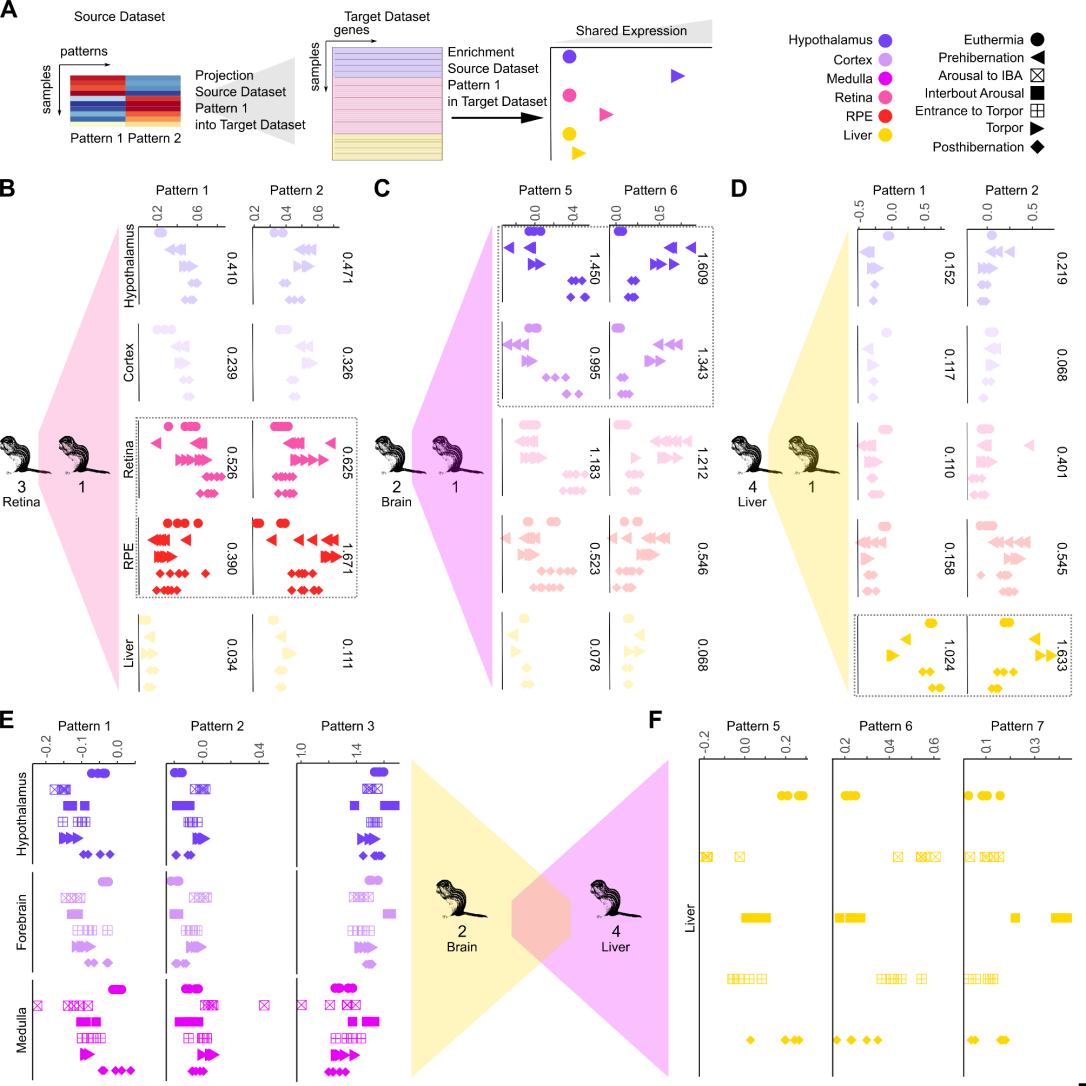
Shared gene expression patterns across datasets. (**A**) Diagram of the procedure of transfer learning to identify shared gene expression. Dot plots of tissue and time point-specific sharing in the 13LGS 1 dataset of patterns from the (**B**) 13LGS 3 (retina), (**C**) 13LGS 2 (brain), and (**D**) 13LGS 4 (liver) datasets. Dashed boxes surround shared and closely-related tissue types. Non-shared tissues are displayed at 25% opacity. Numbers to the right of each plot show the variance within that tissue. (**E**) Dot plots of tissue and time point-specific sharing in the 13LGS 2 dataset of patterns from the 13LGS 4 dataset. (**F**) Dot plots of time point-specific sharing in the 13LGS 4 dataset of patterns from the 13LGS 2 dataset. Each point represents a single sample. RPE = retinal pigment epithelium.

To identify genes with tissue- and state-specificity, we calculated the pattern marker genes for each pattern in each dataset (Supplementary Table 2, listed in order of pattern specificity). Each gene receives a pattern weight for each pattern. A high pattern weight for a gene reflects high expression in the samples enriched for that pattern. A gene may have high pattern weights in multiple patterns (Fig. 3B), but genes that are pattern markers only have high pattern weight in a single pattern (Fig. 3A). The top pattern-specific genes from our analysis aligned with those identified in prior analyses via pairwise comparisons, such as the circadian clock gene *DBP* upregulated in the Chinese alligator hypothalamus in summer^8^ and the transcriptional repressor *TGIF2* upregulated in the torpid heart of the bearded dragon^5^ (Fig. 2).

### Transfer learning reveals shared tissue and time point-specific gene expression across datasets

To determine whether there are shared torpor gene expression patterns across datasets, StrokeofGenus applies the transfer learning R package ProjectR^18^. ProjectR takes as input gene weights from a pattern learned in one dataset and tests for their enrichment in the samples of a second dataset (Fig. 3A). A higher ProjectR score means a sample’s gene expression profile is similar to the tested pattern. If a pattern from a tissue and/or time point in one dataset shows enrichment in the equivalent samples of another dataset, this indicates that the datasets have shared gene expression programs. As proof of principle, we first applied ProjectR to different datasets generated from the same species with overlapping tissues. For this purpose, we used our 13LGS 1 dataset, which profiles multiple tissues, as a scaffold to compare with other 13LGS datasets that profile only one or a few tissues.

We first explored shared time point- and tissue-specific patterns of gene expression between the 13LGS 3 and 13LGS 1 datasets (Fig. 3B, S4C). The 13LGS 3 includes only retinal tissue. Retinal euthermic Pattern 14 and torpid Pattern 11 found in the 13LGS 1 dataset (Fig. S1A), showed significant enrichment in the euthermic and torpid samples, respectively, in the 13LGS 3 dataset (Fig. S4C, Pattern 14 *P* = 3.3e-4, Pattern 11 *P* = 4.6e-3, student’s t-test). Similarly, torpid Pattern 2 from the 13LGS 3 dataset (Fig. S1G) demonstrated the greatest variance in neural-related tissues in 13LGS 1, highlighting the tissue-specificity of this pattern across datasets. It also shows significant separation of torpid samples in the retina (Fig. 3B, torpor/euthermia *P* = 2.5e-3, torpor/3 days post hibernation *P* = 5.7e-3, torpor/14 days post hibernation *P* = 7.7e-3), while the euthermic Pattern 1 showed enrichment in the euthermic Posthibernation retinal samples in 13LGS 1 (Fig. 3B days post hibernation/torpor *P* = 3.7e-3, 14 days post hibernation/torpor *P* = 8.4e-3). We found similar shared gene expression for both 13LGS 2 and 13LGS 4 with 13LGS 1.

We further compared the 13LGS 2 dataset, which contains only brain tissue samples, with the 13LGS 1 dataset (Fig. 3C, S4D). Pattern 6 in the 13LGS 2 dataset, which is enriched in torpid samples (Fig. 2D), showed greater variance within the four neural-related tissues than the liver samples in the 13LGS 1 dataset (Fig. 3C). In the hypothalamus, Pattern 6 showed significant enrichment in torpid samples relative to euthermic time points (torpor/euthermia *P* = 0.014, torpor/3 days post hibernation *P* = 0.031, torpor/14 days post hibernation *P* = 0.022). Similarly, Pattern 5, which is enriched in euthermic samples (Fig. 2D), demonstrated greatest variance in neural-related tissues and showed significant segregation between euthermic Posthibernation time points and torpid time points in the 13LGS 1 hypothalamus samples (Fig. 3C days post hibernation/torpor *P* = 5.1e-4, 14 days post hibernation/torpor *P* = 3.5e-3). Pattern 7 from the 13LGS 1 dataset, which was enriched in the euthermic 14 days post hibernation hypothalamus samples (Fig. S1A), was also enriched in euthermic samples in the hypothalamus of the 13LGS 2 dataset, while the torpid Pattern 5 (Fig. S1A) showed enrichment in the torpid 13LGS 2 hypothalamus samples (Fig. S4D, Pattern 7 euthermia/torpor *p* < 2.2e-16, Pattern 5 euthermia/torpor *p* < 2.2e-16).

The 13LGS 4, which includes only liver tissue, and 13LGS 1 datasets also demonstrate shared gene expression patterns (Fig. 3D, S4E). Pattern 2 from the 13LGS 4 dataset is associated with torpid samples (Fig. 2C). When projected into the 13LGS 1 dataset, this pattern showed greatest variance within liver samples and significant enrichment in torpor liver samples relative to euthermic time points (Fig. 3D, torpor/euthermia *P* = 3.2e-3, torpor/3 days post hibernation *P* = 2.7e-3, torpor/14 days post hibernation *P* = 1.1e-3), consistent with a shared torpor-specific gene expression program. Gene Pattern 1 of the 13LGS 4 dataset is enriched in euthermic time points (Fig. 2C). Projection of pattern 1 into the 13LGS 1 dataset also demonstrated greatest variance in liver samples and significant separation of active liver samples from torpid samples (Fig. 3D, euthermia/torpor *P* = 5.1e-6, 3 days post hibernation/torpor *P* = 2.8e-3, 14 days post hibernation/torpor *P* = 1.8e-3). Though ProjectR identified differences in gene expression between time points in the liver, CoGAPS analysis of the 13LGS 1 dataset only produced a general liver Pattern 20 that did not identify any time point-specific gene expression (Fig. S1A). This demonstrates that ProjectR is able to discern gene expression differences between samples when other sensitive methods are unable to do so. Interestingly, when pattern 20 of the 13LGS 1 dataset was projected into the 13LGS 4 dataset, it was enriched in euthermic time points (Fig. S4E, post hibernation/entrance to torpor *P* = 0.016). These results cumulatively demonstrate that StrokeofGenus can identify shared gene expression programs across multiple datasets generated from different laboratories.

To demonstrate that the functionality of StrokeofGenus is not limited to mammals, we additionally compared two datasets from the Chinese alligator and found shared euthermic and torpor gene expression for each tissue shared between the two datasets (hypothalamus, skeletal muscle, and liver), though the limited number of replicates prevents statistical comparisons (Fig. S4A, B). These results further demonstrate the ability of ProjectR to identify shared gene expression programs despite disparate collection times.

We also found shared gene expression patterns across tissues within the 13LGS. For example, the euthermic brain Pattern 5, torpid Pattern 6, and IBA Pattern 7 found in the 13LGS 2 (Fig. 2D) demonstrate significant enrichment in the matching time points of the 13LGS 4 (Fig. 3E, Pattern 5 euthermia/entrance to torpor *P* = 1.1e-4, Pattern 6 entrance to torpor/euthermia *P* = 1.3e-3, Pattern 7 IBA/entrance to torpor *P* = 6.2e-4). Similarly, projection of euthermic Pattern 1, torpid Pattern 2, and IBA Pattern 3 liver patterns into the 13LGS 2 dataset demonstrated coordinated upregulation in the corresponding time points in the forebrain (Fig. 3F, Pattern 1 euthermia/torpor *P* = 1.1e-4, Pattern 2 torpor/euthermia *P* = 1.6e-4, Pattern 3 IBA/torpor *P* = 2.1e-4). Thus, within the same species, patterns of gene expression in hibernation are shared not only across datasets but also across tissues.

### Torpor gene expression programs are shared across species and different forms of torpor

Transfer learning provides an opportunity to quantify the degree of sharing of torpor gene expression patterns across species and even across forms of torpor. Therefore, we used ProjectR in StrokeofGenus to test for gene expression conservation in the most common tissues across datasets–brain, liver, skeletal muscle, and white adipose–representing different taxonomic groups and forms of torpor.

**Fig. 4:**
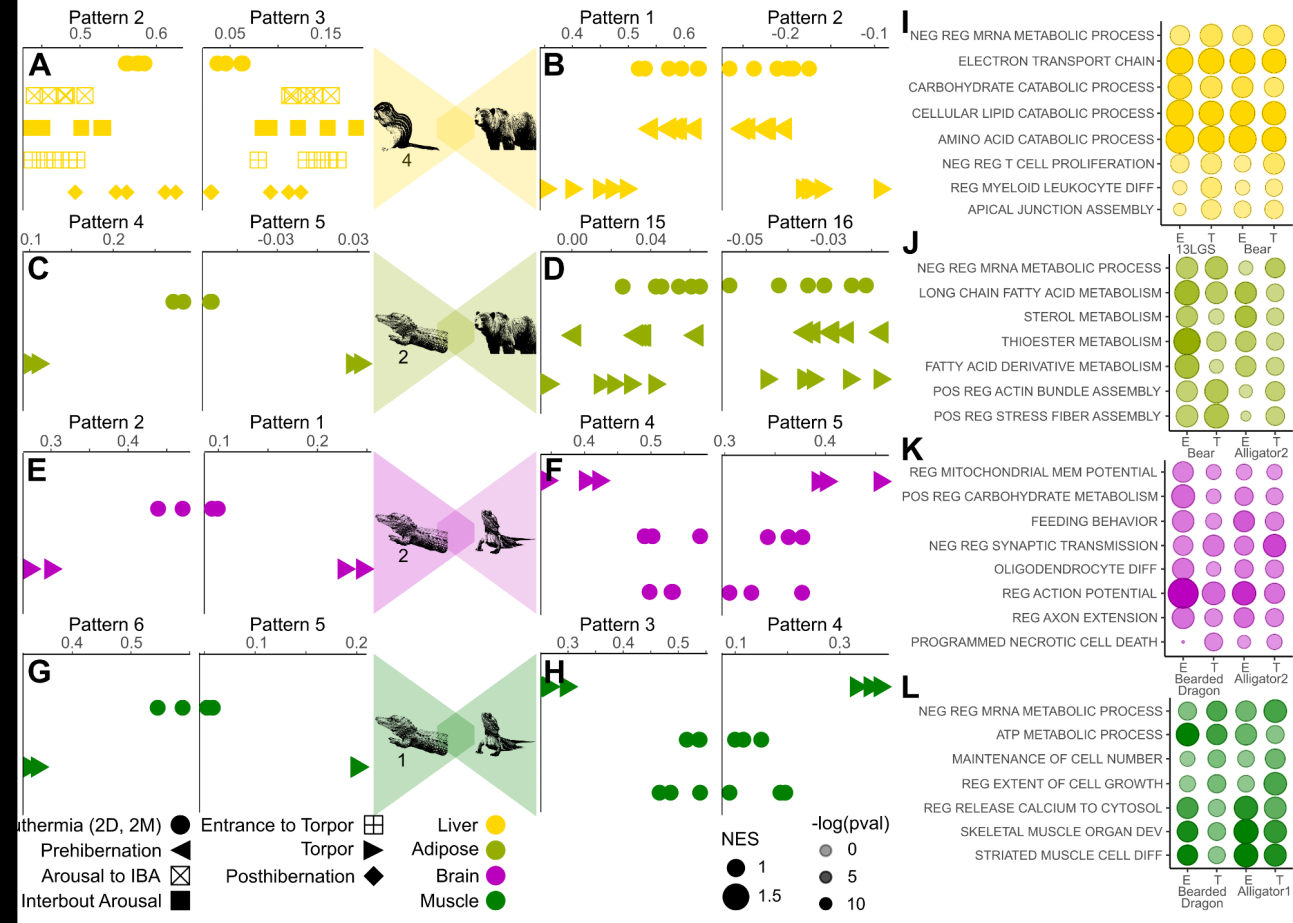
Shared gene expression patterns across species. (**A**) Dot plots displaying time point-specific sharing in the 13LGS 4 dataset of torpor and euthermic patterns from liver samples in the grizzly dataset. (**B**) Dot plots displaying time point-specific sharing in the liver samples of the grizzly dataset of torpor and euthermic patterns from the 13LGS 4 dataset. (**C**) Dot plots displaying time point-specific sharing in the white adipose tissue (WAT) samples of the Chinese alligator 2 dataset of torpor and euthermic patterns from the WAT samples of the grizzly dataset. (**D**) Dot plots displaying time point-specific sharing in the WAT samples of the grizzly dataset of torpor and euthermic patterns from the WAT samples of the Chinese alligator 2 dataset. (**E**) Dot plots displaying time point-specific sharing in the hypothalamus samples of the Chinese alligator 2 dataset of torpor and euthermic patterns from the brain samples of the bearded dragon dataset. (**F**) Dot plots displaying time point-specific sharing in the brain samples of the bearded dragon dataset of torpor and euthermic patterns from the hypothalamus samples of the Chinese alligator 2 dataset. (**G**) Dot plots displaying time point-specific sharing in the skeletal muscle samples of the Chinese alligator 1 dataset of torpor and euthermic patterns from the skeletal muscle samples of the bearded dragon dataset. (**H**) Dot plots displaying time point-specific sharing in the skeletal muscle samples of the bearded dragon dataset of torpor and euthermic patterns from the skeletal muscle samples of the Chinese alligator 1 dataset. Each point represents a single sample. Dot plots displaying the enrichment of biological process gene sets in torpor (T) and euthermic (E) patterns in (**I**) liver in 13LGS 3 and grizzly, (**J**) in adipose in Chinese alligator 2 and grizzly, (**K**) in hypothalamus/brain in Chinese alligator 2 and bearded dragon, and (**L**) in muscle in Chinese alligator 1 and bearded dragon. The X-axis corresponds to the CoGAPS pattern and the Y-axis corresponds to the gene set. Dot size reflects the normalized effect size (NES) and the shade the -log(p-value) of enrichment.

To determine whether we could identify shared torpor gene expression patterns between species, we compared liver torpor expression between the grizzly bear and the 13LGS (most recent common ancestor (MRCA) ~ 90 million years ago (MYA), Fig. S5A), both of which hibernate. Pattern 3 from the grizzly dataset showed significant enrichment in torpid liver samples, whereas Pattern 2 is enriched in euthermic liver samples (Fig. S1C). When projected into the 13LGS 4 dataset, these two grizzly patterns showed significant enrichment in the torpid and euthermic 13LGS samples, respectively (Fig. 4A, Pattern 3 entrance to torpor/euthermia *P* = 3.6e-3, Pattern 2 euthermia/entrance to torpor *P* = 1.8e-4). We performed the reciprocal comparison, projecting the 13LGS 4 dataset into the grizzly dataset, and found that Pattern 2, enriched in torpid time points, and Pattern 1, enriched in euthermic time points (Fig. 2C), showed enrichment in the corresponding grizzly liver samples (Fig. 4B, Pattern 2 euthermia/torpor *P* = 0.016, Pattern 1 euthermia/torpor *P* = 9.2e-4). Pattern markers from the source dataset with orthologs in the target dataset showed matching expression in the target dataset (Fig. S5B, C).

We also found that Pattern 3 and Pattern 2 from the grizzly dataset were enriched in the equivalent time points in bat liver (MRCA ~ 80 MYA, Fig. S5A, S6B), though small sample numbers prevented statistical comparison, and Patterns 4 and 5 in the bat showed equivalent enrichment in the liver samples of the grizzly (Fig. S6A, Pattern 4 euthermia/torpor *P* = 2.0e-8, Pattern 5 torpor/euthermia *P* = 0.014). This demonstrates the shared torpor gene expression across divergent mammalian species.

To identify whether shared torpor gene expression patterns are detectable over greater taxonomic distances, we compared white adipose tissue in the Chinese alligator 2 to the grizzly (MRCA ~ 320 MYA, Fig. S5A). When projected into the Chinese alligator 2 dataset, euthermic adipose Pattern 4 from the grizzly (Fig. S1C) showed enrichment in euthermic adipose samples (Fig. 4C). Similarly, projection of the torpid grizzly Pattern 5 (Fig. S1C) into the Chinese alligator 2 dataset demonstrated segregation between torpid and euthermic replicates (Fig. 4C), though small sample numbers prevented statistical comparisons in either case. Programs associated with euthermic adipose tissue in the Chinese alligator 2 dataset, Pattern 15 (Fig. S1B), show enrichment in euthermic adipose tissue in grizzly (Fig. 4D, euthermia/torpor *P* = 0.012). Taken together, these results suggest shared gene expression programs in torpor across vertebrate classes and hundreds of millions of years of evolutionary divergence.

To determine if distinct forms of torpor also share similar gene expression programs, we compared the Chinese alligator, which enters torpor in response to cold, to the bearded dragon, which enters torpor in response to extreme heat (MRCA ~ roughly 280 MYA, Fig. S5A). Euthermic and torpid brain patterns from bearded dragon showed enrichment in Chinese alligator 2 euthermic and torpid brain samples, respectively, though sample number precluded statistical comparisons (Fig. 4E). Similarly, euthermic and torpid hypothalamus patterns 4 and 5 from the Chinese alligator 2 dataset showed significant enrichment in the euthermic and torpid samples of the bearded dragon brain, respectively (Fig. 4F, Pattern 4 two days post torpor (2D)/torpor *P* = 0.017, two months post torpor/torpor *P* = 0.020, Pattern 5 torpor/2D, *P* = 0.048, torpor/2M *P* = 0.082). Shared gene expression was also found between Chinese alligator brain patterns 2 and 3 and bearded dragon brain, though the separation between time points did not rise to statistical significance (Fig. S6E). Shared torpor-specific gene expression between Chinese alligator and bearded dragon is also apparent in skeletal muscle (Fig. 4G, H, Chinese alligator 1 dataset Pattern 3 2D/torpor *P* = 3.1e-3, 2 M/torpor *P* = 1.2e-4, Pattern 4 torpor/2D *P* = 0.017, torpor/2M *P* = 3.1e-4). We found greater sharing for patterns where conserved genes had greater pattern weight, meaning comparisons can be hampered over large taxonomic distances (Fig. S5D, E). Not only is torpor gene expression shared across species and over large taxonomic distances, but across forms of torpor employed under opposite environmental conditions, such as extreme heat and cold.

We further found that Patterns 1 and 2 from the Djungarian hamster hypothalamus, which undergoes daily torpor, showed time point-specific enrichment in the hypothalamus of Chinese alligator 1, which undergoes brumation, though the limited number of samples precludes statistical analysis (MRCA ~ 320 MYA, Fig. S5A, S6C). Though the Chinese alligator euthermic hypothalamus Pattern 1 did not show significant time point enrichment in hamster hypothalamus, the torpid hypothalamus Pattern 2 was significantly enriched in torpid over euthermic hamster hypothalamus (Fig. S6D, Pattern 2 torpor/euthermia *P* = 0.0276). Torpor gene expression is shared across forms of torpor with very different torpid bout lengths.

To discern which biological processes comprise shared gene expression programs, we applied gene set enrichment analysis (GSEA). We found enriched biological processes that agree with known phenotypes in torpor. Enrichment for gene sets was calculated for gene expression patterns within each species (Supplementary Table 11), then gene sets with similar enrichment were identified across species. As expected, mRNA production was downregulated in torpor across species and tissues^2^ (Fig. 4I, J, L). Metabolic functions were upregulated in euthermic liver for both 13LGS and grizzly^2^ while immune cell development was selectively downregulated in torpid samples^39^ (Fig. 4I). Adipose function was upregulated during euthermia in both grizzly and Chinese alligator 2^2^ (Fig. 4J). As has been found in the Yakut ground squirrel brain^40^, actin fiber assembly was upregulated in both grizzly and Chinese alligator torpid adipose samples (Fig. 4J). Similar to the results in liver and adipose, terms relating to tissue function were enriched in euthermic muscle in Chinese alligator 1 and bearded dragon (Fig. 4L). Torpid animals retain muscle mass despite long periods of inactivity^2^ and in both Chinese alligator 1 and bearded dragon genes controlling maintenance of cell number were upregulated in torpid muscle samples (Fig. 4L). Synapses retract during torpor before being rapidly regrown following a return to euthermia^3^. In both Chinese alligator 2 and bearded dragon brain samples, axon extension is upregulated in euthermic samples (Fig. 4K). Interestingly, oligodendrocyte differentiation is also enriched in the euthermic brain in both Chinese alligator 2 and bearded dragon (Fig. 4K). Demyelination of the hippocampus with the oligodendrocyte toxin cuprizone directs neurons to a dormant, axon-protective state in mice^41^. Not only do diverse species that employ different forms of torpor show shared torpor gene expression programs, but these genes also regulate shared torpor-related biological processes.

Interestingly, metabolic aspects of the form of torpor reflect differences in gene expression. The grizzly bear is a fat-storing hibernator, meaning its white adipose tissue performs lipid catabolism throughout torpor. In contrast, the Syrian hamster is a food-storing hibernator, whose white adipose tissue performs both lipid anabolism and catabolism during torpor. Differences in gene expression in white adipose tissue between food- and fat-storing hibernators has previously been found^12^. To assess whether these gene expression differences constitute programmatic differences, we compared torpor gene expression patterns between Syrian hamster and grizzly bear white adipose tissue (MRCA ~ 90 MYA, Fig. S5A). Though the Syrian hamster euthermic Pattern 1 showed no time point enrichment in the grizzly adipose, the torpid Pattern 3 showed enrichment in euthermic time points over torpid time points (S6F, euthermia/torpor *P* = 0.0041). Similarly, the Grizzly euthermic adipose Pattern 4 shows no time point enrichment in Syrian hamster, but the Grizzly torpid adipose Pattern 5 showed enrichment in euthermic hamster time points (S6G, euthermia/torpor *P* = 0.17, IBA/torpor *P* = 0.0025). In sum, we found opposite pattern enrichment between torpor and euthermic time points in grizzly bear and Syrian hamster white adipose. The Syrian hamster dataset only covers white adipose tissue, so we cannot determine whether gene expression programs are shared between fat- and food-storing hibernators in other tissues.

### Reference genome annotation produces qualitatively equivalent results to de novo transcriptome

The rate of production of high-quality reference genomes for nontraditional model organisms has rapidly increased^42,43^, though some torpor model organisms still lack a reference genome^44–47^. StrokeofGenus includes the functionality to use a reference genome rather than generate a *de novo* transcriptome, which we demonstrate in 13LGS (GCF_016881025.1). An average of 33% more orthologs were identified between 13LGS and classical model organisms using the genome (Table S1). We hypothesize that this is because the genome does not have the same constraints surrounding tissue number and read depth as the *de novo* transcriptome. Nonnegative matrix factorization identified equivalent gene expression patterns for each dataset tested and transfer learning between 13LGS dataset 2 and the grizzly bear liver found similar shared pattern enrichment between genome and *de novo* transcriptome (Fig. 2D, E, Fig. S1G, Fig. S7A-E).

### Artificially-induced torpor demonstrates shared gene expression with natural torpor

Multiple groups have developed approaches to induce torpor in non-heterotherm species^48,49^. Li et al. induced torpor in the MCF7 human cell line by inhibiting the chloride channel cystic fibrosis transmembrane regulator (CFTR) and analyzed the effects of their treatment using RNA-seq. We applied StrokeofGenus to interrogate whether torpor induced by CFTR inhibition phenocopies naturally-occurring torpor.

**Fig. 5:**
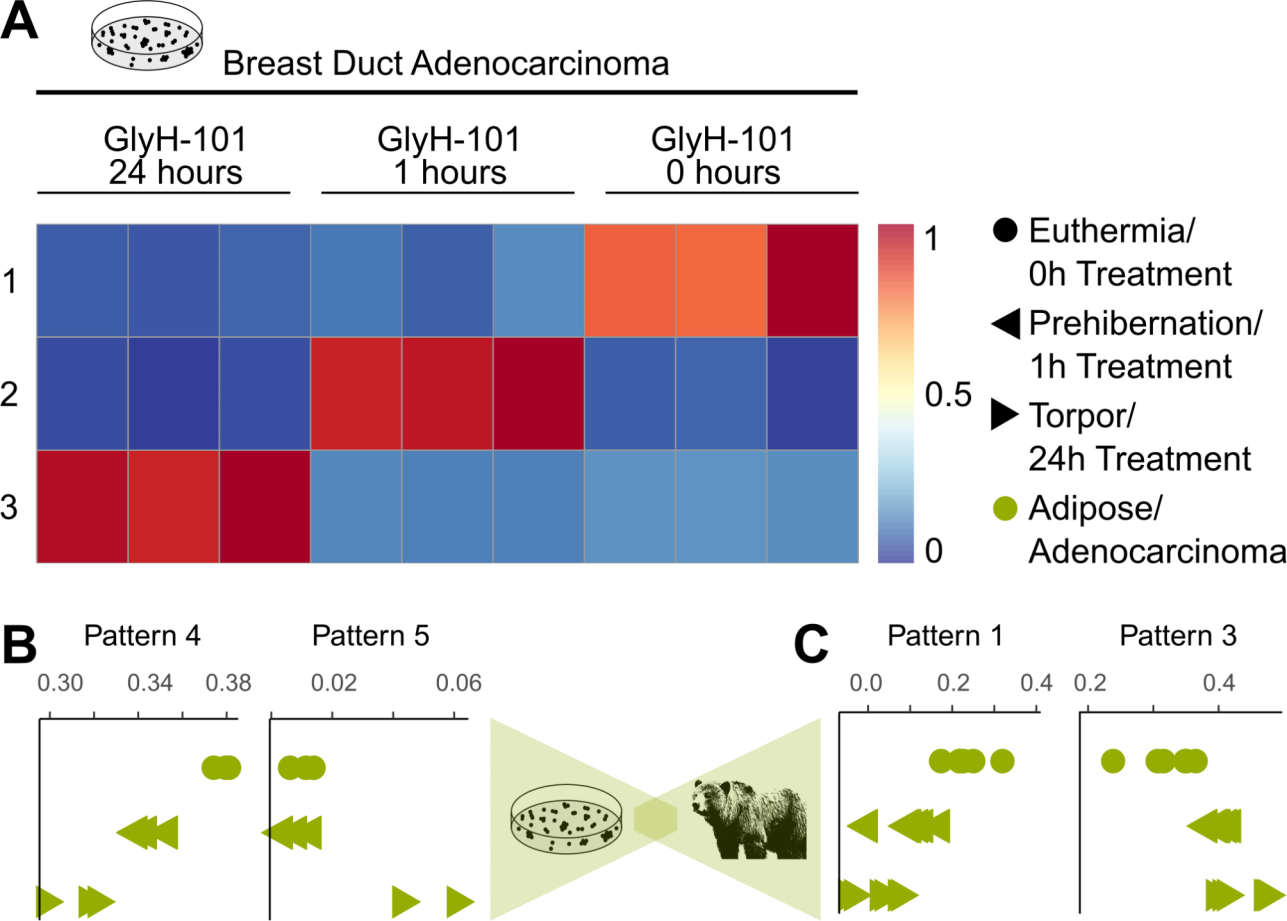
Shared gene expression between artificially-induced and natural torpor. (**A**) Heatmap of treatment-specific gene expression patterns in the MCF7 dataset. Each row is a sample and each column is a CoGAPS-derived pattern. Blue represents low pattern weight and red represents high pattern weight. (**B**) Dot plots displaying treatment-specific sharing in the MCF7 dataset of torpor and euthermic patterns from adipose samples in the grizzly dataset. (**C**) Dot plots displaying time point-specific sharing in adipose samples in the grizzly dataset of torpor and euthermic patterns from the MCF7 dataset. Each point represents a single sample.

We identified three patterns corresponding to untreated, 1-hour, and 24-hour GlyH-101-treated MCF7 samples (Fig. 5A). The MCF7 cell line is derived from breast ductal adenocarcinoma cells^48^. Breast ductal cells derive from stromal adipocyte progenitor cells^50^, so we searched for shared gene expression between the MCF7 dataset and adipose samples from the grizzly dataset. Grizzly Pattern 4, found in euthermic adipose, showed significant enrichment in untreated MCF7 samples over 24 h-treated samples (*P* = 0.0080), and grizzly Pattern 5, found in torpor adipose, showed significant enrichment in 24 h-treated MCF7 samples over untreated (*P* = 0.013) (Fig. 5B). Similarly, MCF7 Pattern 1, found in untreated samples, showed significant enrichment in euthermic over torpid grizzly adipose (Pattern 1 euthermia/torpor *P* = 3.5e-5), and MCF7 Pattern 3, found in 24-hour treated samples, showed significant enrichment in torpid over euthermic grizzly adipose (Pattern 3 torpor/euthermia *P* = 6.5e-5) (Fig. 5C). These findings cumulatively demonstrate that torpor induced by CFTR inhibition in MCF7 cells shows shared gene expression with natural torpor in grizzly adipose, suggesting that CFTR inhibition successfully phenocopies in vivo torpor.

### Limitations of the study

StrokeofGenus relies on the identification of one-to-one gene orthology. This approach simplifies the identification of shared gene expression, but may lead to loss of signal and nuance especially in circumstances of species with genome expansion events.

StrokeofGenus was designed to use *de novo* transcriptomes. In this approach, gene reconstruction and subsequent ortholog discovery are impacted by sample tissue type diversity and sequencing depth. However, StrokeofGenus is also able to use reference genomes which will not be impacted by those same factors.

Shared expression pattern discoverability by StrokeofGenus is heavily dependent on study design. The determination of the correct number of patterns can be impacted when there are limited biological replicates. For instance, the Chinese alligator 2 dataset has only two replicates per condition and some sample-specific patterns were found before all condition-specific patterns were determined (Fig. S1B). Best practice is to generate outputs for a few patterns beyond when the first sample-specific pattern is identified. If more condition-specific patterns resolve, the sample-specific pattern can be disregarded because it is independent of condition-specific patterns and therefore does not impact the identification of condition-specific genes. Confidence in a pattern’s identity can be amplified by including a robust number of biological replicates (Fig. 2, Fig. S1).

Genes that are specifically downregulated in a transcriptomic state can be extracted from pattern weight outputs of CoGAPS. However, CoGAPS does not include a function to specifically generate a list of significant pattern-specific downregulated genes, so users would have to manually extract them from the CoGAPS outputs.

Also, StrokeofGenus has no function to directly determine which genes are driving the sharing of patterns across species. A strong inference can, however, be made by first identifying the global sharing of a pattern in the target dataset, and then identifying pattern markers whose expression in the target dataset aligns with that sharing (Fig. S5B, C). This limitation could be eliminated in the future by the development of computational techniques to discern projection drivers. Overall, however, these limitations of StrokeofGenus are readily surmounted with robust study design.

## Discussion

StrokeofGenus simplifies the analysis of time course gene expression data. Even with expanding numbers of time points, the identification of distinct transcriptomic states and state-specific gene expression is consolidated in a single step without the need for increasingly complex arrangements of pairwise comparisons. The matrix factorization output is also apt for cross-dataset and -species comparisons using transfer learning, enabling the identification of shared gene expression without the need for manual comparison of gene lists.

With the combination of matrix factorization and transfer learning in StrokeofGenus, we identified the distinct transcriptomic states that compose different forms of torpor and demonstrated that some sampled time points are transcriptomically identical. Distinct phases include euthermia, interbout arousal (IBA), and torpor (Figs. 2 and 3). In contrast, we show that pre- and post-hibernation and pre- and post-IBA are indistinguishable from euthermia and torpor, respectively (Figs. 2 and 3). Prior studies have suggested similar conclusions, but the broader view of the transcriptome considered via matrix factorization and transfer learning allows for clearer delineations between transcriptomic states.

Transfer learning demonstrates that torpor gene expression programs for various tissues such as the brain, liver, and white adipose tissue are shared across species, including between the mammal and reptile classes and between forms of torpor such as brumation and aestivation (Fig. 4). Shared gene expression programs, which involve complex inter-regulation of many genes, support the hypothesis that torpor is an ancestral adaptation that has been repeatedly lost rather than repeatedly independently evolved. Further, we found that a torpor phenotype induced in the MCF7 human cell line, which does not naturally undergo torpor, shared gene expression with the torpid grizzly bear, suggesting that non-heterotherms maintain a cryptic torpor phenotype. A prior study demonstrated that heterotherms share regions of accelerated evolution enriched for noncoding regions^51^. Torpor may therefore represent a cryptic metabolic state that only requires mutations in select cis-regulatory elements to be activated. Variations in torpor phenotypes, such as between daily torpor and full hibernation or between fat-storing and food-storing hibernation, may represent activation of different components of this program.

Genes that drive conserved torpor patterns and which are found across multiple species in this analysis are likely to be enriched for important torpor drivers and represent a pool of likely candidates for the manipulation of transcriptomic states. For instance, oligodendrocyte differentiation and axon extension show enrichment in the euthermic brain in both Chinese alligators and bearded dragons (Fig. 4K). Genes directing these processes may play an important role in the brain’s rapid recovery and re-establishment of synapses following torpor^3^.

StrokeofGenus can be applied to further torpor and non-torpor questions. For instance, additional forms of torpor have been described in invertebrate species, such as aestivation in snails and sea cucumbers, the dauer state in nematodes, and diapause in insects^36,47,52,53^. Identification of shared gene expression between torpor in more basal animals and those discussed in this paper could push back the date of the evolution of torpor and generalize the torpor state to include pauses in development. An additional goal of torpor research is to identify effective methods of inducing torpor in non-heterotherm species, which could improve organ transplantation and space flight^54–57^. As demonstrated in this paper, application of matrix factorization and transfer learning can determine how closely induced torpid states match naturally-occurring torpor. Our pipeline could further be applied to identify shared gene expression across species for shared processes other than torpor, such as shifting coat color in response to seasonal changes, post-infection immune recovery, or limb regeneration^58–60^. Any process that involves shifts between transcriptomic states and which is shared across species could be investigated for shared gene expression using matrix factorization and transfer learning.

## Electronic supplementary material

Below is the link to the electronic supplementary material.

Supplementary Material 1

Supplementary Material 2

Supplementary Material 3

Supplementary Material 4

Supplementary Material 5

Supplementary Material 5

Supplementary Material 5

Supplementary Material 8

Supplementary Material 9

Supplementary Material 10

Supplementary Material 11

Supplementary Material 12

## Data Availability

Publicly-available RNA-seq reads were downloaded from the European Nucleotide Archive for each species: 13LGS (PRJNA418486, PRJNA702062, PRJNA361561), Djungarian hamster (PRJNA743775), Australian central bearded dragon (PRJNA476034), grizzly bear (PRJNA413091), Brandt’s bat (SRP017183), monito del monte (PRJNA416414), Syrian hamster (PRJDB6278), and Chinese alligator (PRJNA593416, PRJNA556093), and MCF7 (PRJNA513383). Raw sequencing reads generated for this study will be available upon publication on the Gene Expression Omnibus as GSE248932.
