# Supplementary figures and images for: Identification of shared gene expression programs activated in multiple modes of torpor across vertebrate clades
Source: Sci Rep. 2024 Oct 17;14:24360. doi: 10.1038/s41598-024-74324-5 (PMC11487170; doi:10.1038/s41598-024-74324-5)

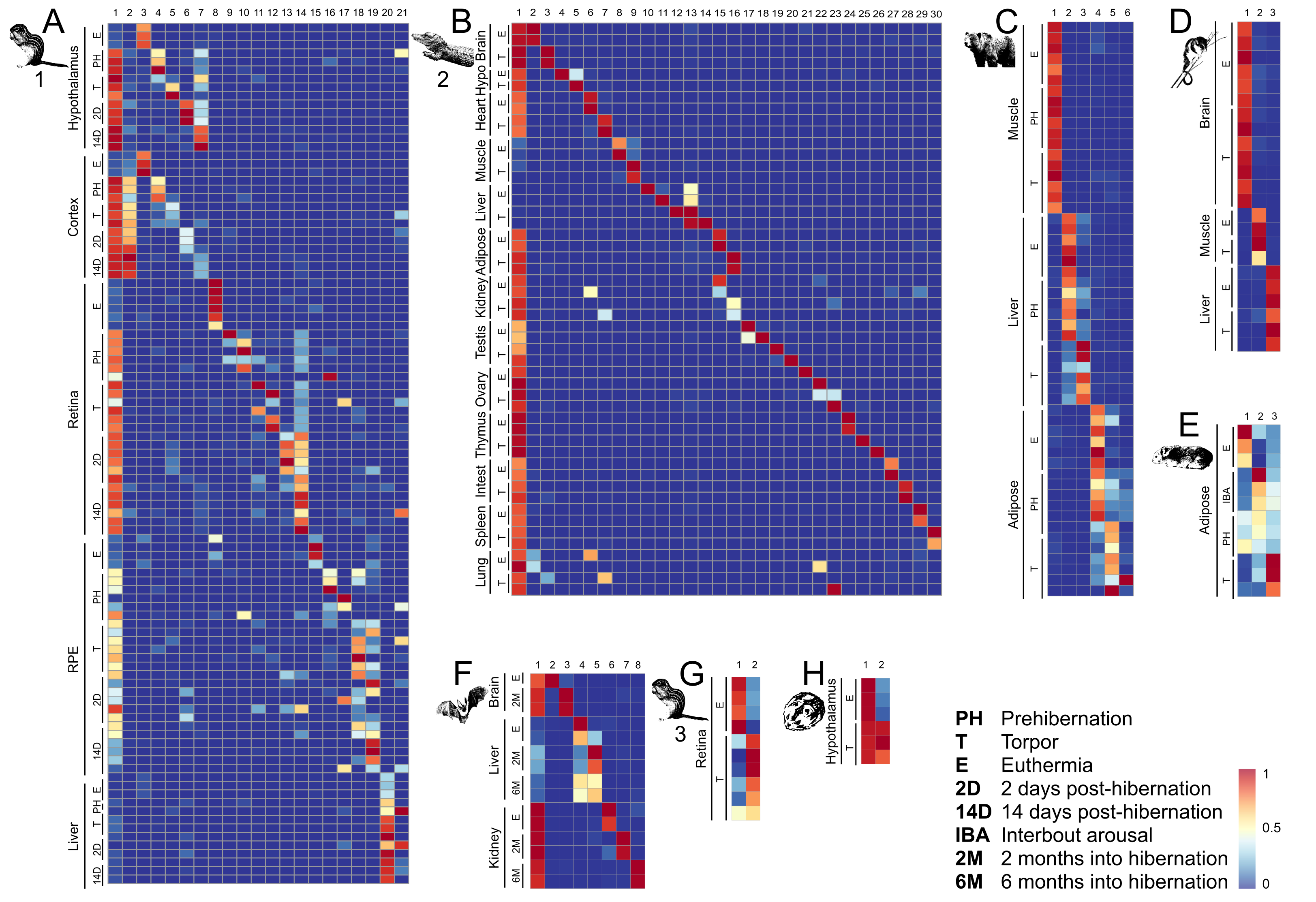

Supplement: Supplementary file 1 — Supplementary Material 1 [file 41598_2024_74324_MOESM1_ESM.png]

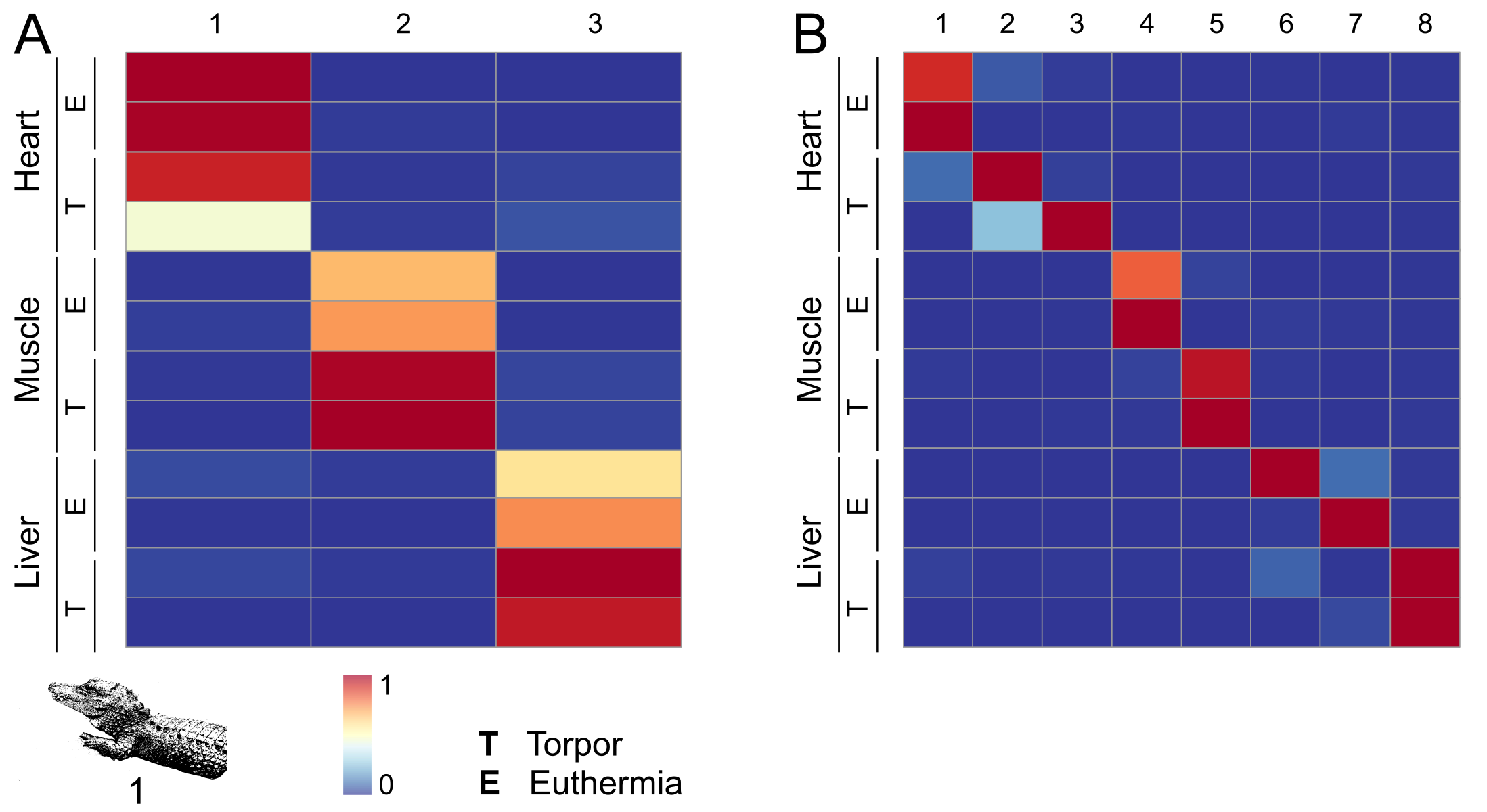

Supplement: Supplementary file 2 — Supplementary Material 2 [file 41598_2024_74324_MOESM2_ESM.png]

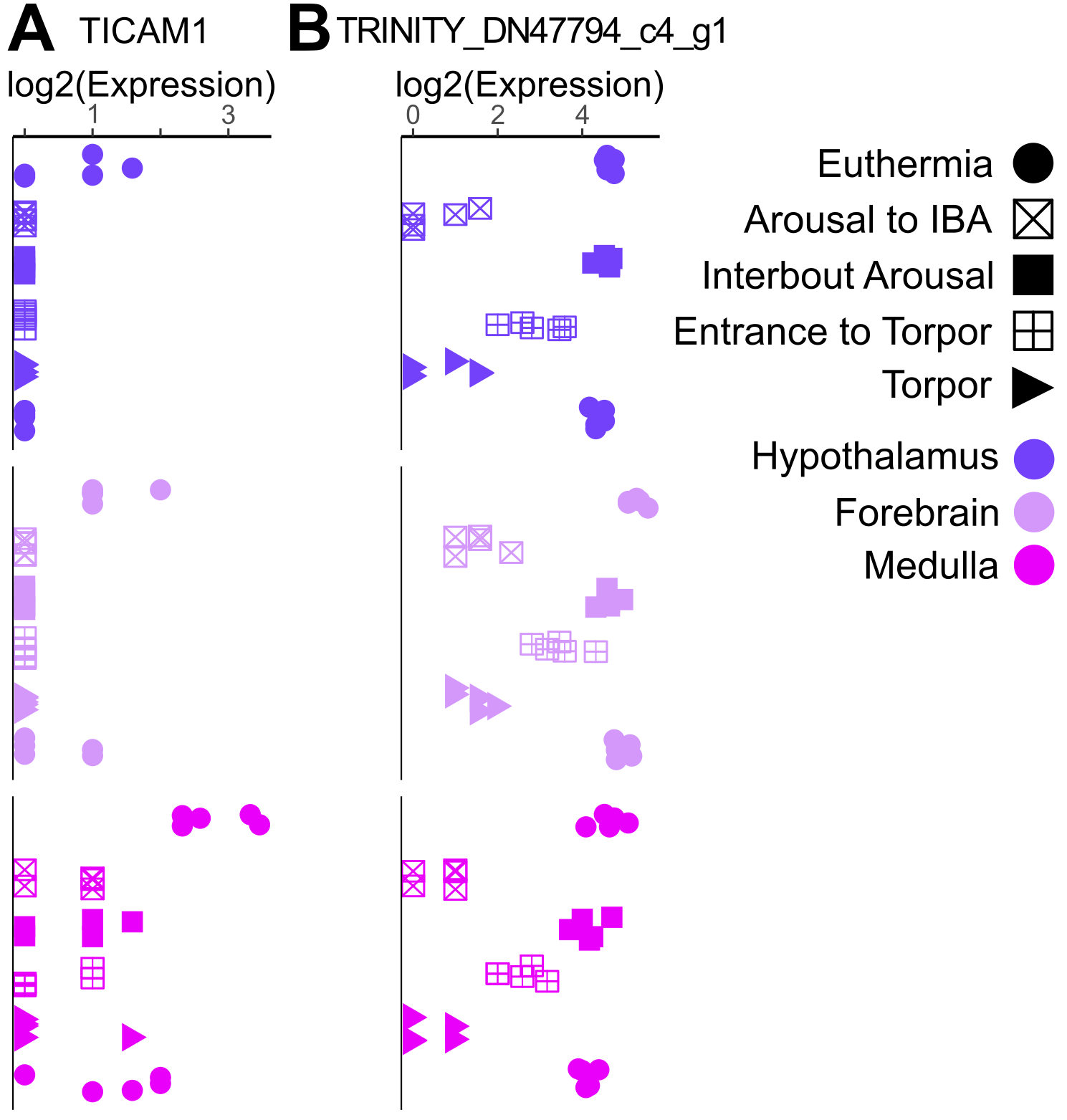

Supplement: Supplementary file 3 — Supplementary Material 3 [file 41598_2024_74324_MOESM3_ESM.png]

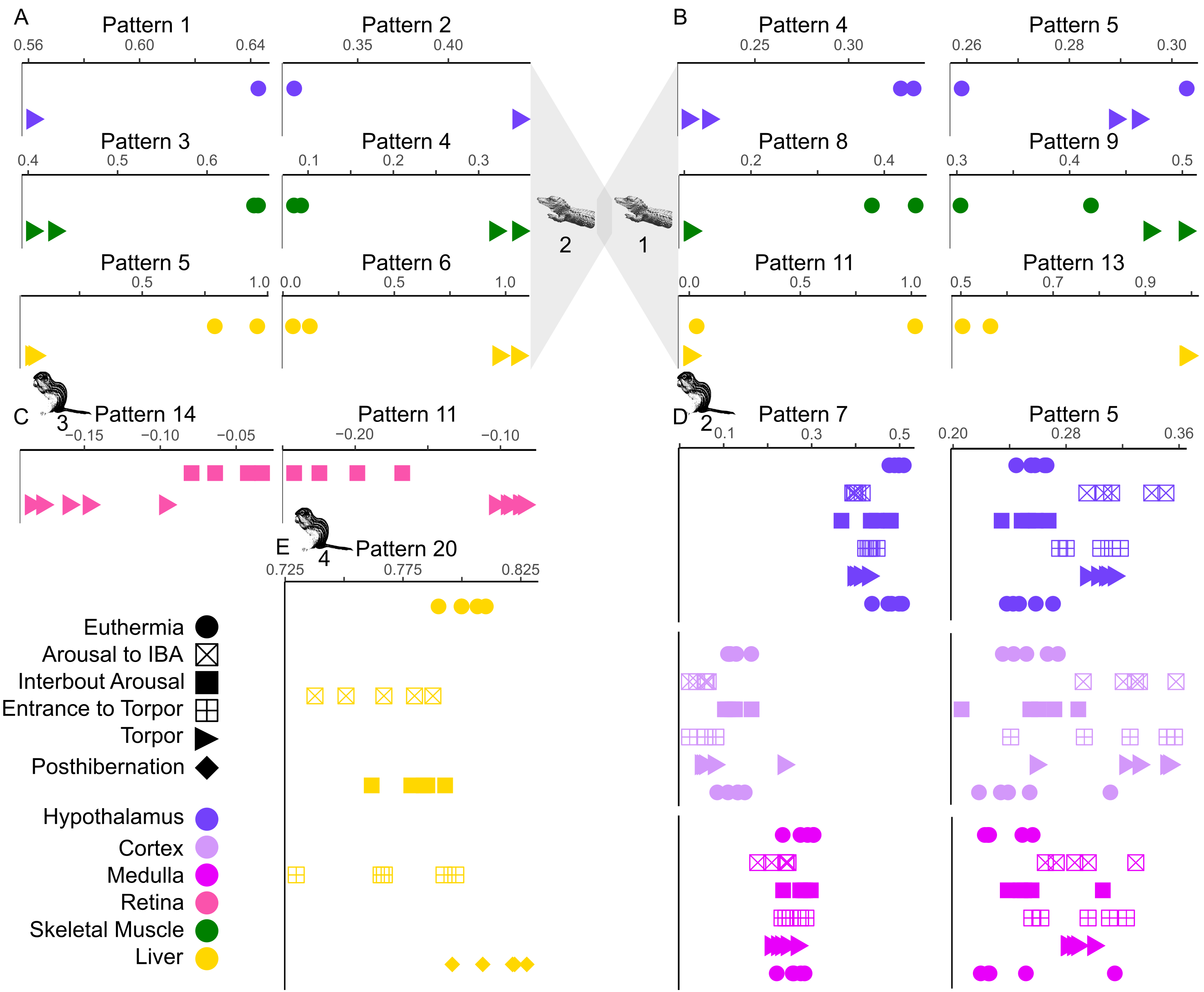

Supplement: Supplementary file 4 — Supplementary Material 4 [file 41598_2024_74324_MOESM4_ESM.png]

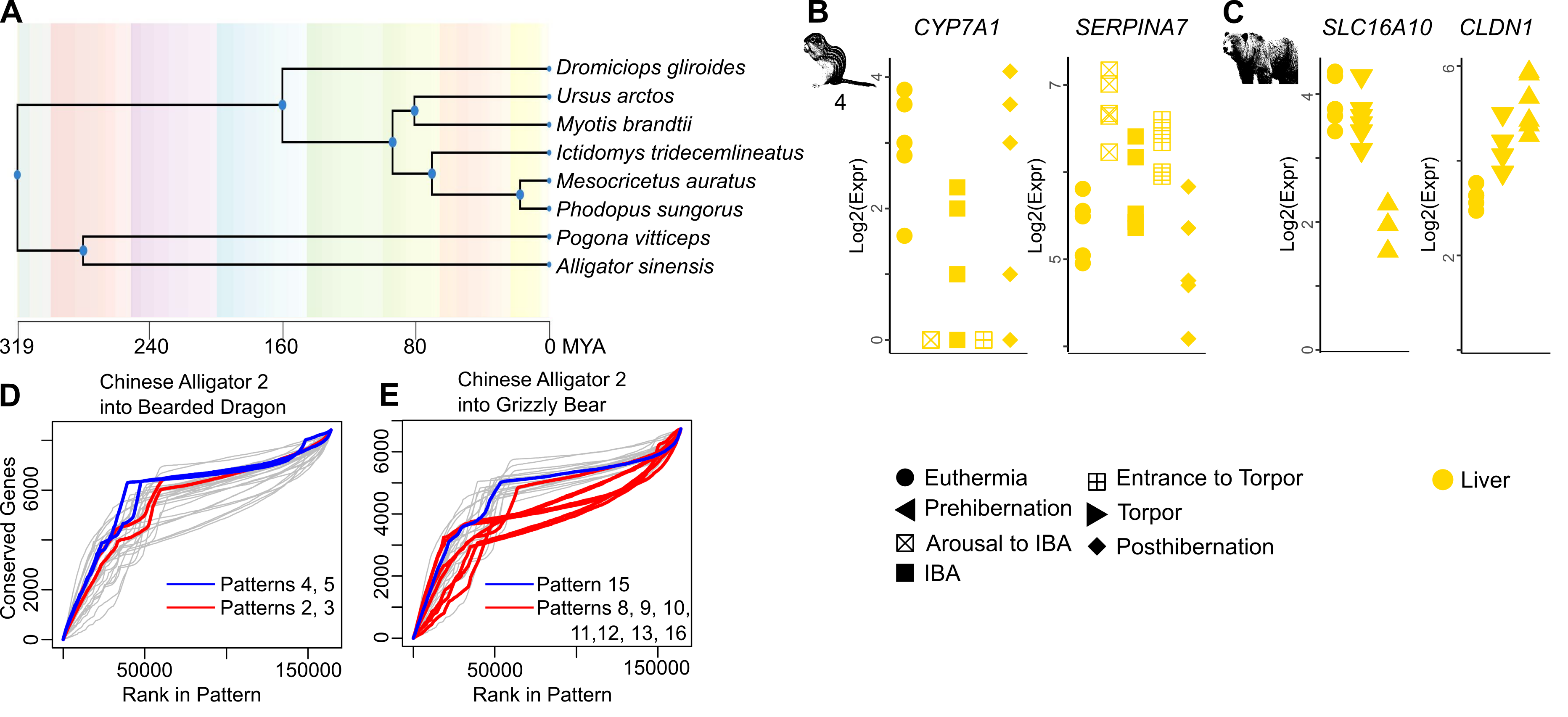

Supplement: Supplementary file 5 — Supplementary Material 5 [file 41598_2024_74324_MOESM5_ESM.png]

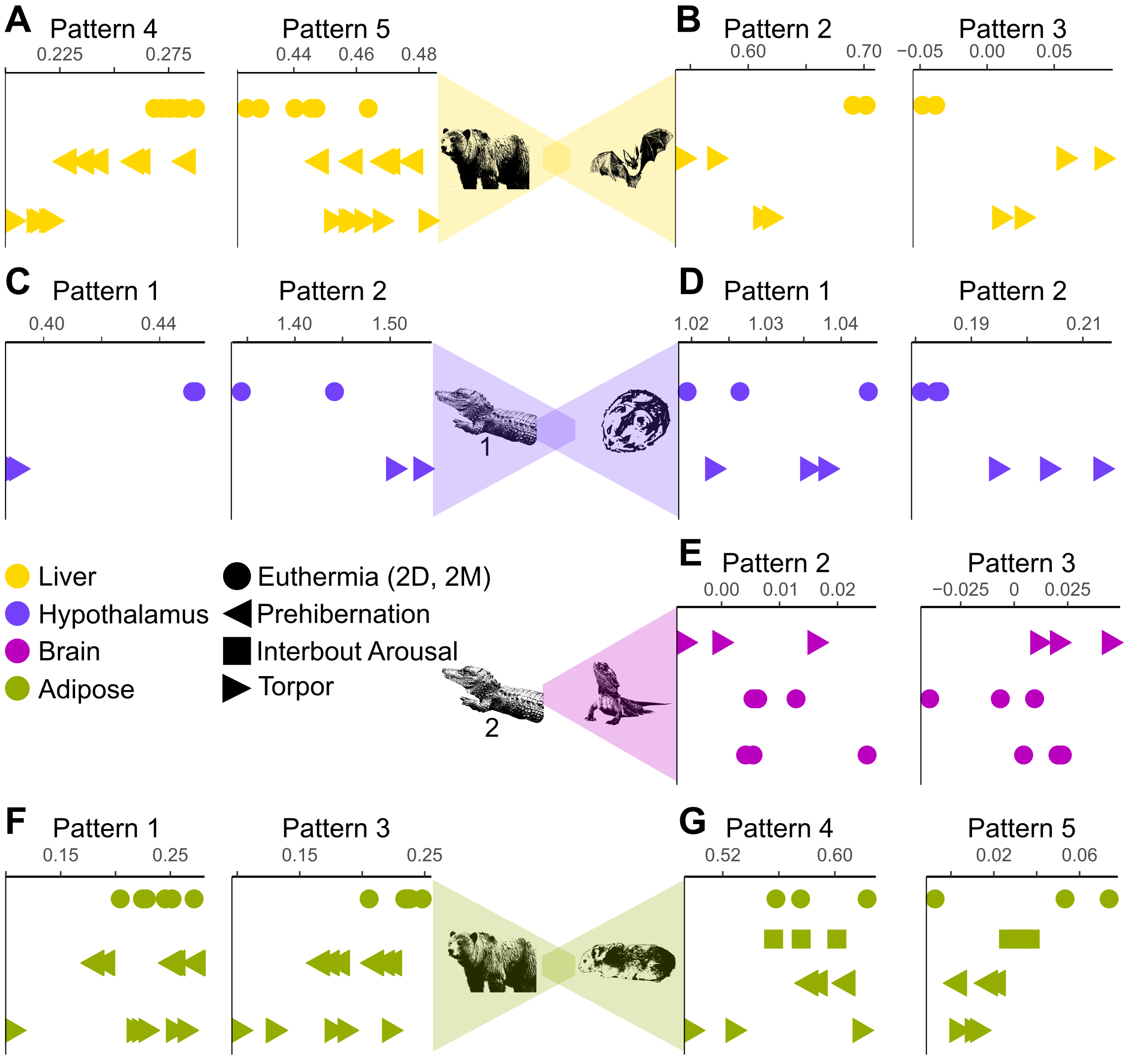

Supplement: Supplementary file 6 — Supplementary Material 5 [file 41598_2024_74324_MOESM6_ESM.png]

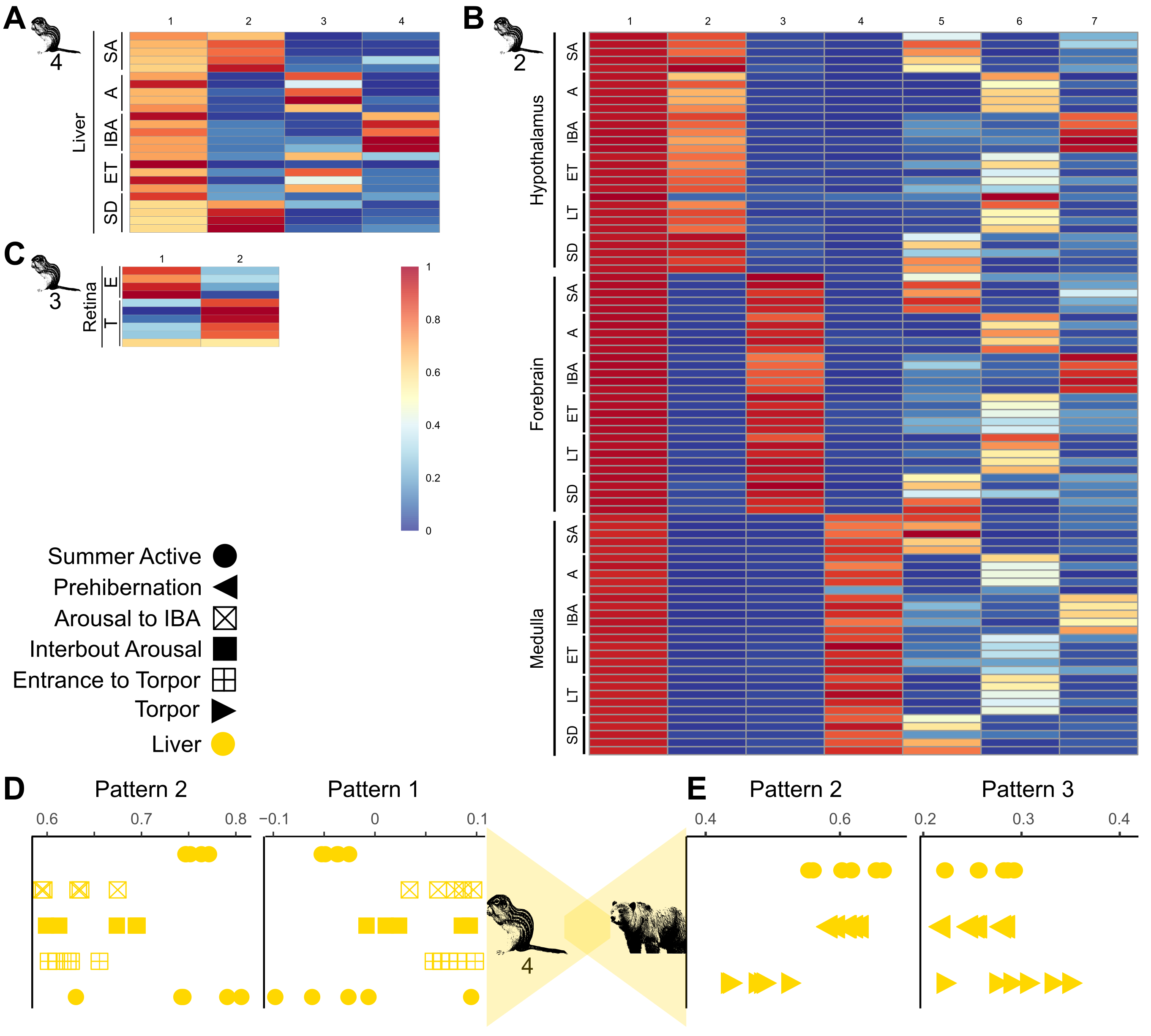

Supplement: Supplementary file 7 — Supplementary Material 5 [file 41598_2024_74324_MOESM7_ESM.png]
