# Supplementary material for: Identification of shared gene expression programs activated in multiple modes of torpor across vertebrate clades
Source: Sci Rep. 2024 Oct 17;14:24360. doi: 10.1038/s41598-024-74324-5 (PMC11487170; doi:10.1038/s41598-024-74324-5)
Supplement: Supplementary file 12 — Supplementary Material 12 [file 41598_2024_74324_MOESM12_ESM.docx]

**SUPPLEMENTAL FIGURES**

**Figure S1**: Identification of tissue and time point-specific gene expression patterns in torpor datasets. Heatmaps of tissue/time point-specific gene expression patterns in the (A) 13LGS 1 dataset, (B) Chinese alligator 2 dataset, (C) grizzly dataset, (D) monito del monte dataset, (E) Syrian hamster, (F) bat, (G) 13LGS 3, and (H) Djungarian hamster. Each row is a sample and each column is a CoGAPS-derived pattern. Blue represents low pattern weight and red represents high pattern weight.

**Figure S2:** Strategy for determining pattern number in a dataset. Heatmaps of the Chinese alligator 1 dataset with (A) three patterns showing tissue-specific gene expression and (B) eight patterns showing either tissue and time point specificity or sample specificity. Each row is a sample and each column a CoGAPS-derived pattern. Blue represents low pattern weight and red represents high pattern weight.

**Figure S3:** Representation of pattern marker genes. Dot plots displaying time point-specific expression of (A) a pattern marker for the euthermic pattern and (B) a gene with high pattern weight in the euthermic pattern but which is not a pattern marker in 13LGS Dataset 2. Each point represents a single sample.

**Figure S4:** Shared gene expression patterns across datasets. Dot plots displaying time point-specific sharing (A) in Chinese alligator 2 of patterns from Chinese alligator 1, (B) in Chinese alligator 1 of patterns from Chinese alligator 2, (C) in 13LGS 3 of patterns from 13LGS 1, (D) in 13LGS 2 of patterns from 13LGS 1, and (E) in 13LGS 4 of patterns from 13LGS 1. Each point represents a single sample.

**Figure S5:** Shared gene expression patterns across species. (A) Dendrogram displaying hierarchical evolutionary relationships between species included in this study. Dot plots showing time point-specific sharing of pattern markers from (B) grizzly in 13LGS 4 and (C) from 13LGS 4 in grizzly. Each point represents a single sample. Survival curves showing the rank for each pattern of the Chinese alligator 2 dataset for genes conserved with (D) bearded dragon and (E) grizzly. Blue lines show patterns with strong sharing across species and red lines show patterns with poor sharing. MYA = million years ago.

**Figure S6:** Shared gene expression patterns across species. Dot plots displaying time point-specific sharing (A) in grizzly liver samples of patterns from the bat dataset, (B) in bat liver samples of patterns from the grizzly dataset, (C) in Chinese alligator 1 hypothalamus sample of patterns from the Djungarian hamster dataset, (D) in Djungarian hamster hypothalamus sample of patterns from the Chinese alligator 1 dataset, (E) in bearded dragon brain samples of patterns from the Chinese alligator 2 dataset, (F) in grizzly adipose samples of patterns from the Syrian hamster dataset, and (G) in Syrian hamster adipose samples of patterns from the grizzly dataset. Each point represents a single sample.

**Figure S7:** Recapitulation of analysis using reference genome. Heatmaps of tissue/time point-specific gene expression patterns in the (A) 13LGS 4 dataset, (B) 13LGS 2 dataset, and (C) 13LGS 2 dataset. Each row is a sample and each column is a CoGAPS-derived pattern. Dot plots of (D) time point-specific sharing in liver samples of the 13LGS 4 dataset of patterns from the grizzly dataset and (E) time point-specific sharing in liver samples of the grizzly dataset of patterns from the 13LGS 4 dataset. Each point represents a single sample.

**Supplementary Tables**

**Table S1.** (Orthology) Similarity matrix showing the number of orthologs identified between each species using Omastandalone. Additional row shows number of orthologs identified with 13LGS reference genome. (Gene Number) Table displaying the number of samples submitted to Trinity for *de novo* transcriptome assembly and the number of genes output by TransDecoder.

**Table S2.** Tables display the pattern markers identified for each pattern in each dataset. The top row describes the samples in the dataset in which the pattern is enriched. The second row describes samples in other species that display apparent sharing of the pattern. Gene names of the human ortholog are used. Genes are listed in descending order of specificity for the pattern in question.

**Table S3.** Table listing the dataset, condition, replicate number, and fastq files for each sample submitted to Trinity for reconstruction of a *de novo* transcriptome for 13LGS.

**Table S4.** Tables displaying enrichment calculated by gene set enrichment analysis of Gene Ontology terms for each pattern within each species. Each page corresponds to one pattern in one species.
